# Are vitamin D and vitamin D receptor levels different in children with developmental dysplasia of the hip?

**DOI:** 10.1186/s13018-020-02162-y

**Published:** 2021-01-07

**Authors:** Duran Topak, Muhammet Seyithanoğlu, Fatih Doğar, Ali Aydın Karadeniz, Burak Tanrıverdi, Fırat Ozan, Ökkeş Bilal

**Affiliations:** 1 Faculty of Medicine, Department of Orthopaedic and Traumatology, Kahramanmaras Sutcu Imam University, Kahramanmaras, Turkey; 2 Faculty of Medicine, Department of Biochemistry, Kahramanmaras Sutcu Imam University, Kahramanmaras, Turkey; 3 Department of Orthopedics and Traumatology, Kayseri Training and Research Hospital, Kayseri, Turkey

**Keywords:** Developmental dysplasia of the hip, Risk factor, Vitamin D receptor, Vitamin D

## Abstract

**Introduction:**

Developmental dysplasia of the hip (DDH) is a common disorder and associated with significant morbidity of the hip joint. Several risk factors have been identified for DDH. The aim of this study is to investigate whether vitamin D and vitamin D receptor (VDR) levels differ in children with DDH and whether they have an effect on DDH development.

**Materials and methods:**

A total of 40 (17 males, 23 females; 9 right hips, 16 left hips, 15 bilateral hips) children who were treated for developmental dysplasia and 40 (23 males, 17 females) healthy children without any musculoskeletal system and metabolic disorders were included in this study between January and June 2019. Blood samples from the DDH and control groups of children were collected to measure the serum levels of vitamin D, VDR, calcium (Ca), phosphorus (P), and alkaline phosphatase (ALP). The levels of Ca, P, and ALP were analyzed using the automated standard spectrophotometric laboratory method. The levels of vitamin D and VDR in the samples were analyzed using enzyme-linked immunoassay.

**Results:**

There were no significant differences in the serum levels of Ca, P, ALP, and vitamin D between the DDH and healthy groups (Ca 9.96 ± 0.47 vs. 9.92 ± 0.48 mg/dL, respectively, *p* = 0.721; P 5.3 ± 0.94 vs. 4.82 ± 0.88 mg/dL, respectively, *p* = 0.23; ALP 252.22 ± 170.15 vs. 245.3 ± 130.93 U/L, respectively, *p* = 0.839). However, serum VDR levels were significantly lower in children in the DDH group (5.77 ± 3.51 ng/mL) than in the healthy control group (9.25 ± 6.43 ng/mL) (*p* = 0.004).

**Conclusions:**

In conclusion, we believe that low VDR levels can affect DDH regardless of the serum levels of Ca, P, ALP, and vitamin D. More comprehensive studies involving parents are needed to understand whether VDR levels mediate genetic transmission in DDH or not.

## Introduction

**Fig. 1:**
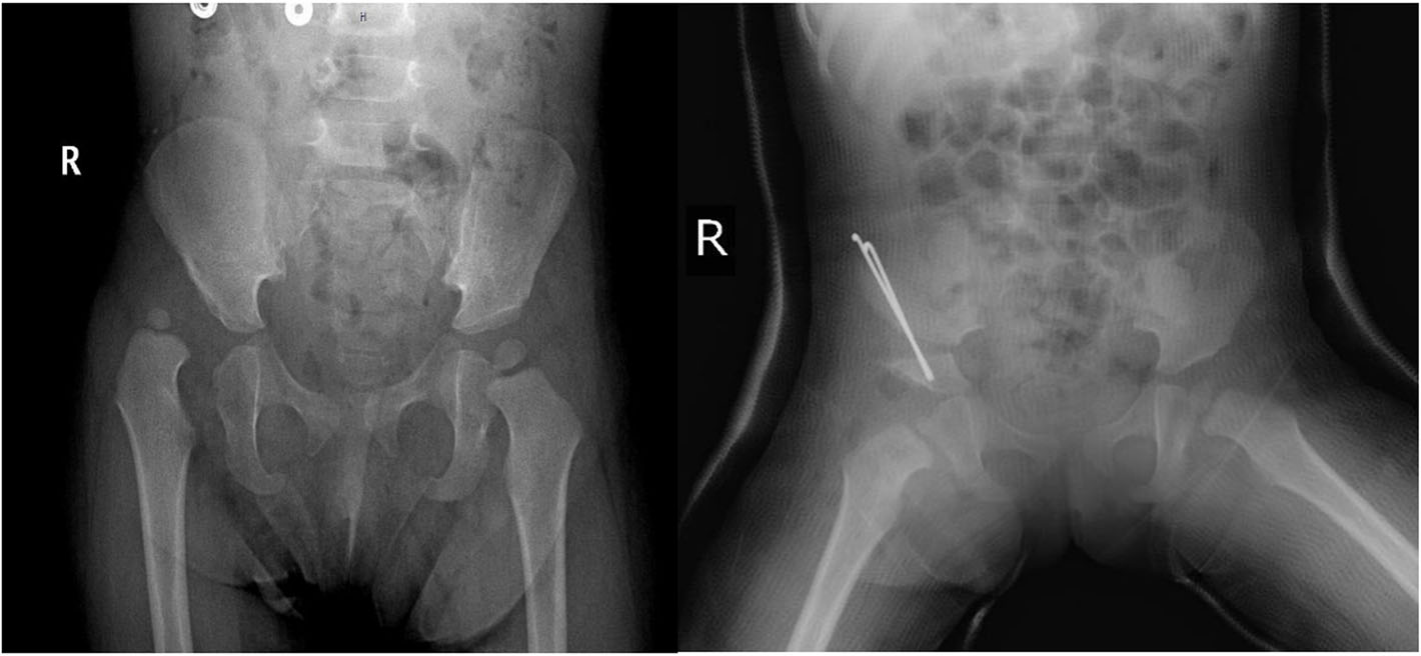
Twenty-one-month-old female, right DDH preoperative and postoperative X-ray images

Developmental dysplasia of the hip (DDH) is a common disorder and associated with significant morbidity of the hip joint [1–3]. Early identification of DDH can provide early correction of the anatomy of the hip, thereby leading to recovery without the need for any major reconstructive surgery (Fig. 1) [1, 2, 4].

The incidence of DDH has been reported to be between 1.6 and 28.5 per 1000 neonates [2]. The majority of children with DDH are females, and the left hip is generally affected, 20% of the cases being bilateral [1–3, 5]. The risk factors for DDH include breech presentation, positive family history, sex, oligohydramnios, prematurity, low birth weight, and amniotic fluid abnormalities [1–3, 5].

The importance of early diagnosis has been confirmed in the treatment of DDH [1, 2]. Currently, DDH screening is performed by clinical examination (Ortholani maneuver, Barlow provocative test, and abduction restriction), ultrasound scanning, or radiography for patients at risk. Although ultrasound has been recommended as a screening test [1, 3], it is not significantly better than at-risk or selective screening [3]. Therefore, it would be highly desirable to identify the predictors of DDH in a high-risk population.

Numerous studies have investigated vitamin D as a risk factor in several diseases [6–11]. In addition to its effects on mineral metabolism, vitamin D exerts its effects on different systems. Vitamin D deficiency has been associated with different diseases such as skeletal system disorders, brain dysfunction, cellular dysfunction, chronic kidney disease, diabetes, disorders of immune response, cancer, abnormalities in the mechanism of insulin release and glucose tolerance, neuromuscular function disorders, and heart failure. Moreover, its association with morbidity, especially in children with DDH, can be significant [3, 6–11]. Vitamin D receptors (VDRs) are found in almost all tissues in the body, and all biological actions of vitamin D are mediated by its binding to VDR [6, 9].

The aim of this study is to investigate whether vitamin D and vitamin D receptor (VDR) levels differ in children with DDH and whether they have an effect on DDH development.

## Materials and methods

Eighty patients were included in the study, which is an observational, prospective, case-control type study. Children of 6 to 90 months old were divided into two groups. A total of 40 (17 males, 23 females; 9 right hips, 16 left hips, 15 bilateral hips) children who were treated for developmental dysplasia (group DDH) and 40 (23 males, 17 females) healthy children (group control) were included in this study from January to June 2019. DDH was diagnosed on the basis of clinical criteria and ultrasound and radiographic examinations depending on the age. Healthy children consisted of patients who applied to the pediatric clinic for routine control, and they did not have any family history of DDH or hip disorders. All of the children in the DDH and control groups consist of children living in the Turkish race and Eastern Mediterranean region. The venous blood of the children in both groups was taken once between eight and ten o’clock in the morning.

The inclusion criteria for this study are as follows: children diagnosed with DDH between 6 and 90 months and completely healthy children without any orthopedic, genetic, and chronic diseases in the same age group. Exclusion criteria for this study are as follows: vitamin D and calcium (Ca) absorption disorders (metabolic bone disease, chronic kidney failure, genetic disorders, epiphyseal dysplasia), children with musculoskeletal anomalies, and children whose parents did not give consent to participate in the study

This research protocol was approved by our local Ethics Committee. Written informed consent was obtained from all subjects before their inclusion in the study (session: 2019/14; date: 31 July 2019; decision no: 11). Informed consent of children was obtained from their families.

### Blood sample collection

The venous blood of the children in both groups was taken once between eight and ten o’clock in the morning. The venous blood taken by the phlebotomy method was placed in tubes without anticoagulants. The serum and plasma were obtained by centrifuging the collected blood for 10 min at 4000 rpm. The serum and plasma obtained were stored at − 80 °C until the working day. On the working day, all samples were brought to room temperature. Serum vitamin D receptor levels were determined using commercial ELISA kit procedures (E-EL-H2043; Elabscience, USA). Measurement of 25-OH vitamin D in the plasma obtained was determined with a UV detector on the HPLC device (Thermo Ultimate 3000, USA) using the Vitamin D ClinRep HPLC kit (Recipe Chemicals Instruments, Munich, Germany). Serum calcium, alkaline phosphatase, and phosphorus levels were measured with original kits of Siemens Advia 1800 (Siemens Healthcare GmbH) biochemistry autoanalyzer.

### Statistical analysis

Statistical analysis was performed using IBM SPSS 21.0 (IBM, Armonk, NY, USA). Frequency analysis was performed for categorical variables. Data were expressed as numbers and percentages. Pearson’s chi-square tests were performed to compare the categorical data. *p* < 0.05 was considered as statistically significant.

Multivariate analysis was performed to compare the dependent variables in two independent groups (MANOVA). In the test, it was seen that the two groups were homogeneously distributed, and the equality was impaired only by VDR levels (*p* = 0.001). In the multivariate test, there was *p* = 0.002 in the Wilks lambda test, and there was a significant difference between the two groups.

## Results

Table 1 shows the characteristics and clinical outcomes of the patients. The mean ages of children in the DDH and healthy control groups were 33.62 ± 22.6 months (range, 6–72 months) and 42.92 ± 21.95 months (range, 8–72 months), respectively (*p* = 0.066). Eight (20%) patients in the DDH group had a family history. There were no significant differences in the levels of Ca, phosphorus (P), and alkaline phosphatase (ALP) between the DDH and healthy groups (Ca 9.96 ± 0.47 vs. 9.92 ± 0.48 mg/dL, respectively, *p* = 0.721; P 5.3 ± 0.94 vs. 4.82 ± 0.88 mg/dL, respectively, *p* = 0.230; ALP 252.22 ± 170.15 vs. 245.3 ± 130.93 U/L, respectively, *p* = 0.839).

**Table 1:** Demographic characteristics and clinical outcomes

	DDH group (*n* = 40)	Control group (*n* = 40)	*p* value
Age (months), mean (range)	33.6 (6–72)	42.9 (8–72)	0.066
Sex, *n* (%)			
Female	23 (57.5)	17 (42.5)	0.180
Male	17 (42.5)	23 (57.5)	
25-OH vitamin D (ng/mL), mean	20	22.6	0.394
Vitamin D receptor (ng/mL), mean	5.7	9	**0.004**
Calcium (mg/dL), mean	9.9	9.9	0.721
Phosphorus (mg/dL), mean	5.3	4.8	0.230
Alkaline phosphatase (U/L), mean	252.2	245	0.839

*DDH* developmental dysplasia of the hip

The mean serum vitamin D levels in the DDH and healthy control groups were 20 ± 14.26 and 22.65 ± 13.36 ng/mL, respectively (*p* = 0.394). The serum VDR levels were significantly lower in children in the DDH group (5.77 ± 3.51 ng/mL) than in the healthy control group (9.25 ± 6.43 ng/mL) (*p* = 0.004).

## Discussion

In this study, we attempted to shed light on the relationship between DDH and levels of vitamin D and VDR. We found that the serum levels of vitamin D and VDR in children with DDH were lower than those in healthy children. Furthermore, there were no significant differences between the DDH and control groups in terms of serum levels of Ca, P, and ALP. To our knowledge, this is the first study to evaluate the relationship between VDR levels and DDH in children in the literature.

Vitamin D is classically responsible for the regulation of calcium metabolism, promoting growth and proper remodeling of bones [5, 6, 9]. However, vitamin D also has autocrine or paracrine effects in other extrarenal tissues such as the skin, prostate, lymph nodes, intestine, breast, pancreas, central nervous system, immune system, placenta, and the circulatory system [6, 8–11]. Because of its complex functions, several studies have investigated the association between the genes and proteins in the vitamin D endocrine system with some diseases [7, 10, 11].

All biological actions of vitamin D are mediated by its binding to VDR. VDRs are found in almost all tissues in the body [9]. It is a major factor in the endocrine system, which regulates calcium metabolism and absorption along with other important cellular functions [12].

DDH exhibits multifactorial and etiopathological features. Family studies conducted on DDH have shown that this disease has a genetic component and complies with the autosomal dominant inheritance pattern [7].

Some studies have investigated the genetic basis of DDH via the VDR gene [3]. The VDR gene has important roles in bone mineralization and vitamin D metabolism. Furthermore, polymorphisms in the VDR gene have been linked to osteoporosis [13]. Granchi et al. found an association between osteoarthritis secondary to DDH and the VDR polymorphism Bsm I. They suggested that the VDR gene is involved in the etiology of DDH [14]. However, Kapoor et al. reported that there were possible genetic associations between DDH and VDR polymorphisms [3]. In contrast, Jawadi et al. demonstrated that there were no associations between VDR polymorphisms and DDH [7].

In the present study, no significant differences were found in the serum levels of Ca, P, ALP, and vitamin D in both the DDH and control groups of children. Although these parameters were within normal limits, it has been demonstrated that mutations associated with the VDR gene may inhibit vitamin D metabolism [3, 6]. Vitamin D exerts its effects by binding to VDR, which is found in various tissues [6]. In this study, serum VDR levels were found to be significantly lower in children with DDH than in the control children.

The fact that the cases were selected from the same region and race was seen as the limitation of this study.

In conclusion, we believe that low VDR levels can affect DDH regardless of the serum levels of Ca, P, ALP, and vitamin D. More comprehensive studies involving parents are needed to understand whether VDR levels mediate genetic transmission in DDH or not.

## Data Availability

The data and materials of patients participating in this study are available to us and will be provided by us upon request.

## Abbreviations

DDH: Developmental dysplasia of the hip
VDR: Vitamin D receptor
Ca: Calcium
P: Phosphorus
ALP: Alkaline phosphatase
VDRs: Vitamin D receptors
